# Patient and Public Involvement of young people with a chronic condition in projects in health and social care: A scoping review

**DOI:** 10.1111/hex.13069

**Published:** 2020-05-05

**Authors:** Femke van Schelven, Hennie Boeije, Veerle Mariën, Jany Rademakers

**Affiliations:** ^1^ Netherlands Institute for Health Services Research Utrecht The Netherlands; ^2^ Department of Family Medicine Care and Public Health Research Institute (CAPHRI) Maastricht University Maastricht The Netherlands

**Keywords:** adolescent, chronic disease, disabled children, empowerment, impact, involvement, patient participation, scoping review, young adult

## Abstract

**Background:**

The involvement of young people with a chronic condition in research and implementation projects in health and social care receives growing attention. Yet, there is a lack of conceptual clarity of this so‐called ‘Patient and Public Involvement’ (PPI) and methods to systematically evaluate it are absent. This scoping review aimed to gain insight into developments in the existing literature on PPI of young people with a chronic condition by mapping reported definitions, goals, activities, experiences and impact.

**Methods:**

We conducted searches in Cinahl, Embase, PsycINFO, PubMed and Scopus. Included articles described involvement of young people with a chronic condition in research and implementation projects, contained empirical data, were written in English and were published after 1990. Two researchers independently carried out the data extraction.

**Results:**

Twenty‐three studies out of 4993 initial hits met the inclusion criteria. We found great variation in definitions and operationalizations of PPI. Reflections of authors on the process of PPI and its impact were similar and did not change over the years.

**Discussion and conclusion:**

Limited progress in the evidence base of the impact of PPI with young people with a chronic condition was found. Over the years, studies continue to report similar experiences and challenges. In order to move forward, we suggest future research to make connections to existing work instead, to include thorough descriptions of what is understood by PPI and how this is translated into activities, and to use systematic and objective, but also flexible, methods to measure its impact.

## 1 INTRODUCTION

Internationally, there is growing attention for the involvement of young people with a chronic condition (YPCC).^1^, ^2^ Researchers and policy makers increasingly aim to carry out research projects (eg scientific projects aimed at increasing knowledge) and implementation projects (eg practice‐oriented projects aimed at developing, for example, tools and interventions) together *with* rather than *about* or *for* them.^3^ This is often referred to as ‘Patient and Public Involvement’ (PPI) and is associated with the ratification of the United Nations Convention on the Rights of the Child (UNCRC) in 1989.^4^ According to this convention, all young people have a right to have a say in matters that affect them, without discrimination and irrespective of disabilities.

Young people with a chronic condition's involvement in research and implementation projects in health and social care is also motivated by the expected benefits. Researchers have argued that PPI improves the relevance and quality of projects^1^, ^5^, ^6^ and contributes to the personal development of YPCC.^1^, ^6^, ^7^, ^8^, ^9^ Consequently, there appears to be a general consensus that YPCC’s involvement should become an integral and standard element of projects that affect them.^2^, ^8^, ^10^

However, two systematic reviews have found remarkably little (high quality) evidence concerning the impact of PPI with YPCC.^1^, ^2^ The authors of the first review, published in 2004, have suggested that this was due to the novelty of the research area.^2^ Ten years later, the authors of another review concluded that evidence was still limited.^1^ According to them, this can be related to inadequate reporting and the absence of methods to assess PPI processes and outcomes.

Highly relevant in the discussion about reporting and assessing the impact of PPI is the lack of clarity about the concept.^11^, ^12^, ^13^, ^14^ A well‐known definition of young people's involvement in projects has been formulated by Hart^15^: *the process of sharing decisions which affect one's life and the life of the community in which one lives*. This definition, and other definitions that have frequently been referred to,^3^, ^16^ are rather broad. It has been suggested that these *umbrella definitions* create space for diverse interpretations and meanings of the concept.^11^, ^13^ Consequently, researchers and policy makers have used it in a breadth of ways,^13^, ^14^ making the concept rather unclear.

There is a dearth of research addressing the conceptualization of PPI with YPCC. In 2017, Educational Action Research has dedicated a special issue to conceptualizing impact of PPI,^17^ but the studies in this issue have focused on other or broader populations (eg adults or young people in general). It is suggested that the concept of PPI with YPCC can be somewhat different.^18^

Conceptual clarity about PPI with YPCC is needed to generate more and higher quality evidence concerning its impact.^11^ The current lack of clarity has resulted in the use of different definitions for the same terms, leading to confusion in what exactly is measured.^12^ A more general consensus of what is understood by PPI and what outcomes can be expected is essential in developing methods to systematically assess its impact.

The aim of this review was to gain insight into how PPI with YPCC in projects in health and social care is conceptualized in the existing literature and possible shifts herein. We have mapped reported definitions and goals, activities, experiences and impact. The following research questions were studied:

1. What definitions and goals of PPI with YPCC are described in the existing literature?
2. How are these definitions and goals operationalized in involvement activities?
3. What are the experiences with and impact of these involvement activities?
4. What developments in PPI with YPCC can be seen in the existing literature after the UNCRC was ratified in 1989?

Since this is an emerging field in research, a scoping review design was chosen for the study.^19^, ^20^ This design allows for a flexible and broad approach in mapping research activity, that is summarizing the range of evidence to gain insight in its breadth and depth. From this, conclusions can be drawn regarding the overall state of the literature and research gaps can be identified.

## 2 METHODS

A scoping review was conducted following the stages of the methodological framework developed by Arksey and O’Malley,^19^ refined by Levac, Colquhoun and O’Brien.^20^

### 2.1 Stage 1: Identifying research questions and aim

The questions and aim that guided the review are specified in the introduction. A wide approach was chosen to generate breadth of coverage in this area of research. Important parameters were: young people, that is people aged 12‐25; chronic conditions, that is conditions ‘that last or are expected to last twelve or more months and result in functional limitations and/or the need for ongoing medical care’^21^; and PPI.

### 2.2 Stages 2 and 3: Identifying studies and study selection

The search strategy was developed by the first author with the help of a librarian. It employed variations and Boolean connections (AND, OR) of the following terms: young people, chronic conditions and PPI. Searches were conducted in five databases: Cinahl, Embase, PsycINFO, PubMed and Scopus. Table 1 shows the search string used in PubMed as an example. The last search was performed 23 January 2019.

**Table 1:** Search string in PubMed

Search	Query
#5	Search (((#1 AND #2 AND #3))) AND ("1990"[Date ‐ Publication]: "3000"[Date ‐ Publication])
#4	Search (#1 AND #2 AND #3)
#3	Search ("Stakeholder Participation"[Mesh] OR "Community Participation"[Mesh] OR “Patient participation”[Mesh] OR "Community‐Based Participatory Research"[Mesh] OR research particip*[tiab] OR participatory research[tiab] OR participatory action research[tiab] OR project particip*[tiab] OR program particip*[tiab] OR policy participat*[tiab] OR meaningful particip*[tiab] OR research involv*[tiab] OR patient participation[tiab] OR user involvement[tiab] OR participative[tiab] OR participatory[tiab] OR engagement[tiab] OR collaborative[tiab] OR advocacy[tiab])
#2	Search ("Chronic Disease"[Mesh] OR “Disabled Persons”[Mesh] OR chronic disease*[tiab] OR chronic ill*[tiab] OR chronically ill[tiab] OR chronic condition[tiab] OR chronic disab*[tiab] OR disabled person[tiab] OR physical disab*[tiab] OR physically handicapped[tiab] OR physically challenged[tiab] OR disablilit*[tiab] OR handicapped[tiab] OR physically disabled[tiab] OR mentally disabled[tiab])
#1	Search ("Adolescent"[Mesh] OR "Young Adult"[Mesh] OR adolescen*[tiab] OR young adult*[tiab] OR young people[tiab] OR young person*[tiab] OR youth[tiab] OR teen*[tiab] OR youth[tiab])

#### 2.2.1 Inclusion and exclusion criteria

Studies were included in the review, if they:

1. … addressed YPCC;
  - Young people being defined as people aged 12‐25;
  - Chronic condition being defined as conditions ‘that last or are expected to last twelve or more months and result in functional limitations and/or the need for ongoing medical care’.^21^
2. … reported on PPI in research or implementation projects.
3. … contained empirical data.
4. … were written in English.
5. … were published after 1990 (the year after the ratification of the UNCRC).

Studies that reported on involvement of representatives of YPCC, such as caregivers or care providers, or did not clearly distinguish YPCC as a subgroup were excluded. Studies containing non‐empirical data (eg editorials), literature reviews, meta‐analyses and conference papers were also excluded.

#### 2.2.2 Study selection

One researcher (FS) performed the search and removal of duplicates. She also conducted the initial screening of titles and abstracts and discarded obviously irrelevant studies. Two reviewers (FS and VM) independently screened the remaining titles and abstracts, and subsequently the full texts. Discrepancies were resolved and if necessary a third reviewer (HB) was consulted to make a final decision. The researchers regularly met to discuss challenges and uncertainties in study selection.

The reference lists of included studies were screened to identify additional relevant studies.

### 2.3 Stage 4: Charting the data

A data charting form was developed to extract relevant data from the included studies. The form was piloted on three studies and adapted to ensure it was comprehensive. Two researchers (FS and VM) extracted data on: the study (study aim); the project in which YPCC were involved (project aim); and PPI (definitions and goals, YPCC involved, activities, and outcomes and reflections on PPI processes).

#### 2.3.1 Quality assessment

Arksey and O’Malley^19^ have argued that quality assessment is not part of a scoping review. However, according to Levac et al,^20^ this is debatable. Assessing the quality of the vast range of studies in a scoping review helps to put the results in context and facilitates interpretation. Therefore, we decided to report on the quality of the included studies.

Qualitative studies were assessed using the Critical Appraisal Skills Program (CASP),^22^ which contains ten criteria on study design, recruitment strategy, data collection and analysis, the relationship between researcher and participants, ethical considerations, description of the findings and the value of the overall study. Quantitative studies were assessed using the NIH Quality Assessment Tool for Observational Cohort and Cross‐Sectional Studies.^23^ This checklist covers fourteen criteria concerning study participants, power analysis, timing between exposure and outcome, definition of exposure and outcome measures and presence of bias. Mixed methods studies were screened using the Mixed Methods Appraisal Tool (MMAT),^24^ which contains five specific criteria for qualitative, randomized controlled trial, non‐randomized trial, descriptive and mixed methods designs.

### 2.4 Stage 5: Collating, summarizing and reporting results

Extracted data were analysed using quantitative analysis and qualitative analysis. The quantitative analysis focused on characteristics of the included studies. The majority of the results were analysed qualitatively and focused on descriptions and operationalizations of PPI, outcomes and reflections on PPI processes. The first author developed initial themes based on discussions with all members of the research team.

## 3 RESULTS

### 3.1 Included studies

**Figure 1:**
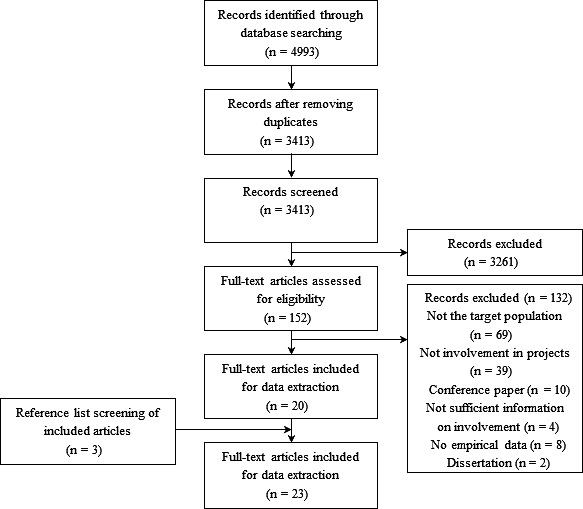
Flow diagram of the article selection process

A total of 4993 studies were retrieved through database searching. After the removal of duplicates and screening of records, twenty‐three studies were included in the review (Figure 1). Nineteen were qualitative,^5^, ^7^, ^9^, ^25^, ^26^, ^27^, ^28^, ^29^, ^30^, ^31^, ^32^, ^33^, ^34^, ^35^, ^36^, ^37^, ^38^, ^39^, ^40^ two quantitative^41^, ^42^ and two mixed methods.^43^, ^44^ In fifteen studies, PPI with YPCC was the outcome of interest.^5^, ^7^, ^9^, ^25^, ^28^, ^29^, ^30^, ^31^, ^32^, ^35^, ^37^, ^38^, ^39^, ^40^, ^42^ In eight studies, it was applied as study method.^26^, ^27^, ^33^, ^34^, ^36^, ^41^, ^43^, ^44^ None of the studies were published before 2000, eight between 2001 and 2010,^5^, ^7^, ^25^, ^29^, ^30^, ^39^, ^41^, ^42^ and fifteen between 2011 and 2018.^9^, ^26^, ^27^, ^28^, ^31^, ^32^, ^33^, ^34^, ^35^, ^36^, ^37^, ^38^, ^40^, ^43^, ^44^

Table 2 provides an overview of the content of the projects in the included studies and the YPCC involved. The studies reported on nineteen unique projects. One project was addressed in two studies,^27^, ^28^ and three studies described findings from multiple projects.^7^, ^30^, ^42^ Of the nineteen unique projects, nine were research projects,^27^, ^28^, ^29^, ^31^, ^32^, ^33^, ^36^, ^38^, ^39^, ^41^ six were implementation projects,^25^, ^26^, ^35^, ^37^, ^40^, ^43^ and four combined elements of both research and implementation.^5^, ^9^, ^34^, ^44^ The studies addressing multiple projects provided results on involvement in implementation projects.^7^, ^30^, ^42^

**Table 2:** Participatory projects in the included studies and the YPCC involved

References	Research or implementation	Participatory project	Young people (YP) involved
Brown et al^25^	Implementation	Designing a switch‐based device to provide control of a Virtual Learning Environment	6 YP (age 16‐19) with physical disabilities
Bruce and Parker^9^	Research & implementation	Preparing advocates to shape the future for themselves and for others	6 YP (age 18+) who are deafblind
Castensoe‐Seidenfaden et al^26^	Implementation	Developing an app to support self‐management	37 YP (age 16‐26) with diabetes
Chappell et al^28^	Research	Studying how issues as love, relationships, sex and HIV/AIDS are discussed in the construction of the sexual sense of self	3 YP (age 15‐20) with physical impairments
Chappell^24^	See Chappell et al^28^		
Coyne et al^43^	Implementation	Co‐developing an e‐health intervention to support transition to adult health care	17 YP (age 15‐23) with CHD, cystic fibrosis and diabetes
Flicker^29^	Research	Improving living conditions	27 YP (age unknown) living with HIV
Franklin and Sloper^30^	Implementation	Not provided (study addresses multiple participatory projects)	YP (age 5‐18) with disabilities
Graham et al^31^	Research	Studying experiences of play	1 YP (age 19) with cerebral palsy
Kramer et al^44^	Research & implementation	Evaluating the extent to which a project was useful and enjoyable	6 YP (age 12‐17) with disabilities
Lightfoot and Sloper^7^	Implementation	Not provided (study addresses multiple participatory projects)	YP (age 13‐20) with arthritis, asthma, cancer, chronic pain, cystic fibrosis, diabetes, eczema, renal failure and spina bifida
Marshall et al^32^	Research	Exploring attitudes and practices in relation to sexual health	7 YP (age 17‐26) with intellectual disabilities
McAnuff et al ^33^	Research	Designing feasible and practicable research in participation outcomes and interventions	6 YP (age 11‐18) with neurodisabilities
Moreau and Eady^34^	Research & implementation	Exploring involvement in medical education	17 YP (age and chronic condition unknown) were interviewed about (desired) involvement in medical education + 12 young people (age and chronic condition unknown) were involved in conducting the study
Murray^35^	Implementation	Facilitating involvement in high level, strategic Children Service's Planning	27 YP (age 5‐25) with learning and physical disabilities and sensory impairments
Powers et al^41^	Research	Obtaining information about transition experiences considered effective and opportunities to participate in them	YP (age 16‐28) with deafness, learning disability, traumatic brain injury, cerebral palsy, epilepsy, blindness, spinal cord injury, bipolar disorder, autism, down syndrome, spinal muscular atrophy, spina bifida and dwarfism (number unknown)
Rahi et al^36^	Research	Identifying content and generating items for a self‐report instrument assessing quality of life	18 YP (age 10‐16) with visual impairments
Rich et al^37^	Implementation	Creating a forum to explore areas of interest; making recommendations to enhance quality and quantity of practice; informing hospital employees; and providing advocacy opportunities and experience	18 YP (age 14‐21) (chronic condition unknown)
Rosen‐Reynoso et al^5^	Research & implementation	Providing postsecondary education transition support	15 + YP (age 16+) (exact number and chronic condition unknown)
Sloper and Lightfoot^42^	Implementation	Not provided (study addresses multiple participatory projects)	YP (age 4‐21, most were 12‐18) with single condition (eg asthma, autistic spectrum disorders, cancer, cystic fibrosis, diabetes, renal failure and sickle cell disease) or multiple conditions
Stevenson^38^	Research	Assisting in achieving life goals and greater social connection	3 YP (age 18‐25) with Down Syndrome
Van Staa et al^39^	Research	Exploring self‐care competencies and preferences	9 YP (age 15+) with dermatologic disorders, blood disorders, neuromuscular diseases, renal failure and diabetes mellitus
Vindrola‐Padros^40^	Implementation	Distilling key findings from a study into an information leaflet	23 YP (age 7‐14) with long term conditions

The included studies reported on PPI of one to over 50 YPCC. YPCC were aged 4‐28 years; five studies (also) included data on YPCC younger than 12^30^, ^35^, ^36^, ^40^, ^42^ and three on YPCC older than 25.^26^, ^32^, ^41^ Six studies focused on young people with one specific condition,^9^, ^26^, ^29^, ^31^, ^36^, ^38^ such as diabetes, renal failure and cystic fibrosis, but most addressed several conditions or a general group of conditions,^7^, ^25^, ^27^, ^28^, ^30^, ^32^, ^33^, ^35^, ^39^, ^40^, ^41^, ^42^, ^43^, ^44^ such as physical impairments or learning disabilities. In some studies, YPCC were included based on additional criteria, other than age and chronic condition, such as previous participatory experiences, educational level and skills and abilities.

#### 3.1.1 Quality assessment

Looking at the quality of the qualitative studies, only three articles met all criteria of the CASP^7^, ^29^, ^34^ and one study met all but one criterion.^9^ The other qualitative studies mostly provided limited or no information on study design and/or methods. In some articles, a section on study methodology or a clear description of methods were absent. Also, they rarely provided a critical reflection on the author's own role in the study, even when YPCC and professionals had a double role of being both the researcher and the researched and were investigating a participatory process they were part of themselves.

The two quantitative studies provided descriptive statistics. Consequently, only the first five criteria concerning the statement of the research aim and the (selection of) study participants of the NIH Quality Assessment Tool were applicable. One study met all applicable criteria.^42^ The other study met all but one, due to convenience sampling of study participants.^41^

The two mixed methods studies did not meet the criteria of the MMAT on representativeness of the quantitative sample and nonresponse bias.^43^, ^44^ Information about the recruitment of participants was missing or a convenience sample was used. One study also lacked methodological information about the qualitative component of the study.^44^ In this study, criteria on deriving and interpreting the qualitative findings were not met, but the small qualitative component had minimal bearing on the overall findings.

### 3.2 The concept of PPI

#### 3.2.1 Terms and definitions

In most studies, the terms participatory research (eg participatory action research, community‐based participatory research, youth‐based participatory research),^5^, ^9^, ^27^, ^28^, ^29^, ^32^, ^34^, ^38^, ^39^, ^41^ participative or participatory design,^25^, ^26^ or user involvement (eg service‐user involvement, patient involvement)^7^, ^33^, ^39^, ^42^ were used for PPI with YPCC. Sometimes more general terms were applied, such as participation,^30^, ^35^, ^44^ participatory approach^40^, ^43^ or advocacy.^31^ One study reported on child‐centred methods,^36^ and one on emancipatory disability research.^38^

An explicit definition of PPI was provided in twelve studies (Table 3).^5^, ^9^, ^28^, ^29^, ^30^, ^31^, ^32^, ^38^, ^39^, ^41^, ^42^, ^43^ In the other studies, PPI was not defined, but two studies described a framework^44^ or key principles.^40^ None of the studies reporting on participative or participatory design explained these terms, the other terms were defined in various ways. The definitions provided did not depend on the terms used, and no differences were found in older or more recent publications.

In the definitions, five recurring elements were identified. First, PPI is a collaborative approach,^5^, ^29^, ^39^, ^43^ meaning that projects are carried out with or by YPCC (not on them).^28^, ^39^ Another element is that participatory projects address topics that matter to YPCC.^9^, ^29^ The third element is that PPI is a continuum of activities in which YPCC have different levels of influence,^28^, ^29^, ^30^, ^42^ varying from being informed to being the main decider.^30^ Allowing YPCC to play a role in various stages of a project is the fourth element we identified.^5^, ^28^, ^39^, ^41^ The last element is that PPI is meaningful to both the YPCC involved and YPCC in general.^5^, ^31^, ^38^, ^41^

We discovered three key principles on which PPI is built. A prominent one is sharing power.^28^, ^29^, ^32^, ^43^ Participatory processes depend on shared decision making, co‐learning and mutual ownership of project products.^39^, ^43^ Another principle is iterative development.^38^, ^40^, ^43^ PPI requires repeated cycles of planning, acting, observing and reflecting.^38^ The third key principle is that participatory processes focus on YPCC’s strengths and resources.^9^, ^29^

#### 3.2.2 Goals

Improving relevance and quality of projects were reported as important goals of PPI.^5^, ^7^, ^9^, ^26^, ^29^, ^33^, ^34^, ^36^, ^39^, ^40^, ^41^, ^42^, ^44^ Other goals of participation are providing YPCC with opportunities for learning and personal development^26^, ^34^, ^41^ and recognizing them as social agents with a unique perspective.^27^, ^28^, ^32^, ^34^ Finally, several studies cited the UNCRC,^7^, ^27^, ^28^, ^30^, ^35^, ^39^, ^42^ suggesting that YPCC’s rights were also a reason for involvement.

### 3.3 Operationalizations of PPI

#### 3.3.1 Recruitment

Several studies have described YPPC’s involvement in the recruitment of other young people. YPCC participated in establishing recruitment processes,^32^, ^34^ designing recruitment materials^7^, ^29^, ^31^ and identifying and recruiting YPCC.^5^, ^27^, ^41^ They advised, for example, on recruitment strategies,^7^, ^32^ chose incentives for research participation,^32^ commented on information sheets for research participants,^31^ distributed flyers^5^ and recruited YPCC from their network.^5^, ^41^

#### 3.3.2 Design

YPCC played a role in designing research,^5^, ^7^, ^29^, ^31^, ^32^, ^33^, ^34^, ^41^ and tools and interventions.^5^, ^25^, ^26^, ^33^, ^40^, ^43^, ^44^ In research projects, they were involved, for example, in drafting research questions^29^ and protocols.^29^, ^31^ In implementation projects, they co‐designed an app for self‐management,^26^ an online curriculum to improve transition to postsecondary education,^5^ and an information leaflet on shared decision making in care.^40^

Studies have reported several design activities with YPCC. Most activities were group consultations, such as participatory workshops^25^, ^26^, ^29^, ^40^, ^43^ or group or panel meetings.^5^, ^7^, ^32^, ^33^, ^34^, ^41^, ^44^ Design activities were seldom individual.^31^, ^40^ Also, virtually all consultations were face‐to‐face.^5^, ^7^, ^25^, ^29^, ^34^, ^40^, ^41^, ^43^, ^44^ One study consulted a mail panel for feedback on a self‐management app.^26^ During consultations, YPCC provided feedback on research designs or prototypes of practical tools or interventions developed by adult professionals,^5^, ^25^, ^26^, ^32^, ^33^, ^40^, ^43^, ^44^ or they developed (parts of) research designs or prototypes themselves.^7^, ^26^, ^41^, ^44^ In some cases, creative techniques were applied, such as an ‘interactive storyboarding process’,^25^ a ‘diamond ranking exercise with images and sorting cards’,^40^ sketching prototypes^26^ and creating video self‐portraits.^26^

#### 3.3.3 Collecting and analysing data

During data collection, YPCC were involved in establishing data collection protocols,^29^, ^32^ and developing surveys and topic guides for interviews and focus groups.^5^, ^7^, ^27^, ^28^, ^34^, ^36^, ^37^, ^39^, ^41^, ^44^ YPCC developed data collection tools themselves (supported by adult researchers),^7^, ^37^, ^39^, ^41^, ^44^ or reviewed and revised materials developed by adult researchers.^32^, ^36^ They also (co‐)conducted surveys, interviews and focus groups.^7^, ^27^, ^28^, ^37^, ^38^ They did this mostly by themselves (supported by adult researchers).^7^, ^27^, ^28^, ^37^, ^38^, ^39^, ^41^ One study mentioned the use of ‘Participatory Rural Appraisal techniques, such as drawing and timelines’, to help YPCC to initiate interview and focus group conversations.^27^

During data analysis, YPCC were involved in performing analyses^27^, ^28^, ^29^, ^38^ and interpreting findings.^5^, ^7^, ^31^, ^32^, ^34^, ^38^, ^39^, ^41^ They were predominantly involved in qualitative analyses, through member checking sessions^31^, ^32^, ^34^, ^38^, ^41^ and meetings in which they discussed and coded interview transcripts.^27^, ^28^, ^29^, ^38^ Other activities included keeping reflexive journals that helped to reflect on other YPCC’s views^27^, ^28^ and a workshop to translate findings into practical guidelines.^7^ In two studies, YPCC were involved in quantitative analyses: they entered responses in a database, calculated average ratings and analysed bar graphs and frequencies,^44^ and reviewed and interpreted findings.^41^

#### 3.3.4 Dissemination

During dissemination, YPCC were involved in writing (research) reports, for example, as co‐authors of a scientific article,^28^, ^44^ or reviewers of articles written by adult researchers.^9^, ^29^, ^39^ Furthermore, YPCC commented on dissemination strategies,^32^ (co‐)presented at, for example, conferences,^7^, ^29^, ^32^, ^35^, ^38^, ^39^, ^41^ launched a website,^43^ created magazines and newsletters^29^ and videos,^5^, ^7^ participated in media activities^39^ and developed a research brief and agenda.^41^

#### 3.3.5 Support

YPCC are provided with different types of support. They participated in trainings on research methods^27^, ^28^, ^39^, ^44^ and advocacy.^9^ Professionals also aided YPCC by providing them with feedback and by discussing solutions for possible issues they experienced.^28^, ^41^, ^42^ Payment^7^, ^29^, ^42^ and gift cards^37^, ^43^ were used to acknowledge them for their time and efforts, and pizza parties and informal meals were organized to create an informal and comfortable atmosphere.^5^, ^29^, ^37^ Funding and support were also provided for transport.^29^, ^32^, ^37^, ^42^

In some cases, professionals working with YPCC also received support, in the form of training or information and additional time.^42^ Some studies reported structural support for youth participation by hiring a person with designated responsibility for PPI^37^, ^42^ or by providing all new personnel with an introduction course on PPI.^30^

### 3.4 Impact of PPI

#### 3.4.1 Note on outcome measures

Patient and Public Involvement was the outcome of interest in some studies and a study method in other studies. In studies in which PPI was the outcome of interest, involvement was the focus of the study. Consequently, these studies have paid extensive attention to its process and impact, and generally have studied and described this more systematically than studies that applied PPI as a study method. In the studies that applied PPI as a study method, authors mostly made a short statement in the discussion about their experiences concerning the process and impact.

#### 3.4.2 Impact on projects

Over the years, mainly positive outcomes of PPI were reported. In general, studies stated that YPCC were able to provide new insights from their lived experience^7^, ^28^, ^35^, ^37^, ^42^, ^44^ and that their involvement positively influenced at least some aspects of projects.^5^, ^9^, ^26^, ^28^, ^29^, ^30^, ^31^, ^32^, ^34^, ^36^, ^38^, ^39^, ^41^, ^42^ Especially data collection and analysis^28^, ^29^, ^31^, ^34^, ^36^, ^41^ and dissemination of project results seem to benefit from YPCC’s involvement,^5^, ^29^, ^32^, ^39^, ^41^, ^42^ but improvements were also reported for research design,^5^, ^29^, ^41^ recruitment^5^, ^29^ and project products, such as an intervention or an article.^9^, ^26^ Limited specific examples were provided contributions of YPCC to projects. PPI increased the relevance of research questions^5^ and usability of practical tools.^26^ Other examples included improved accessibility of consent forms and questionnaires,^32^ better questions in questionnaires^36^, ^41^ and increased media attention.^39^

In some articles, the benefits were somewhat nuanced. Interviews and focus groups conducted by YPCC can lack depth, due to limited skills of asking follow‐up questions.^27^, ^39^ In data collection, YPCC tend to interpret findings assertively, adding their own experiences.^38^ Also, PPI does not necessarily facilitate the recruitment of other YPCC.

#### 3.4.3 Impact on stakeholders

Both older and more recent studies have reported that PPI can also benefit the YPCC involved. Reported benefits included learning new knowledge and skills,^5^, ^28^, ^29^, ^30^, ^37^, ^38^, ^41^ developing confidence,^9^, ^30^, ^37^, ^42^ building new relationships,^7^, ^30^, ^35^ feeling valued^7^, ^29^, ^30^ and learning about themselves.^27^, ^28^ One study reported about PPI as an ‘intensive community development interventions with a strong dose‐response effect’, because it provides YPCC with an opportunity to actively engage in society.^29^

Given the reported benefits, it is not surprising that studies have reported commitment and enthusiasm among YPCC regarding their PPI. YPCC felt proud^32^ and privileged,^38^ and expressed the wish to continue to advocate for their peers in the future.^9^, ^30^, ^38^ Two studies have reported similar feelings among professionals; it increased the commitment of researchers to their research project^39^ and contributed to feelings of inspiration and pride.^29^

### 3.5 Reflections on PPI

#### 3.5.1 YPCC’s motivations

A variety of YPCC’s motivations for PPI were reported, including the wishes to help others and contribute to society,^7^, ^9^, ^29^, ^37^ share their perspectives,^7^, ^34^, ^39^ meet others,^29^, ^39^ learn new skills,^39^ have fun^39^ and do something new.^7^

#### 3.5.2 Time and funding

Both older and more recent studies have shown that participatory projects require additional resources compared with non‐participatory projects, since they develop at a relatively slow pace and are time‐consuming for YPCC and professionals.^5^, ^9^, ^26^, ^29^, ^30^, ^32^, ^37^, ^39^, ^40^, ^42^ Additional funding is also a key enabler of PPI.^7^, ^26^, ^29^, ^30^, ^35^ Lack of time and funding can hinder an ongoing and detailed dialogue between both parties,^7^, ^26^, ^30^, ^35^, ^38^ and result in feelings of frustration and lack of control.^5^, ^9^, ^29^

#### 3.5.3 Representativeness

Over the years, several studies have raised concerns about the representativeness and diversity of the YPCC’s involved in projects. Most participatory projects only include a small number of YPCC.^30^ Also, PPI may require specific qualities and capabilities.^9^, ^30^, ^31^ Studies have suggested that the YPCC involved are more outgoing, critical and self‐confident than their non‐involved peers^34^, ^37^, ^39^ and that ‘hard‐to‐reach’ YPCC are provided with less opportunities for PPI.^30^ Some studies have described a risk of dropping out, due to the process being too strenuous,^9^, ^39^ YPCC losing interest^39^ or being afraid of stigmatizing or losing respect from peer groups.^29^ Consequently, only a ‘core group’^29^ or ‘elite’^39^ may remain involved. For two studies, representativeness was not necessarily the aim of PPI in their project,^33^, ^39^ but ‘to work with service users as a part of a team’.^33^

#### 3.5.4 What activities work?

Studies have reported that YPCC should be involved in all phases of a project,^26^, ^44^ and especially during the first project phases.^7^, ^26^, ^32^ This enables YPCC to participate before research questions or tool design are set in stone.^26^, ^33^

It is suggested that there is ‘not one right method for involvement’.^7^ Appropriate activities vary according to the needs and interests of the YPCC involved and the purposes of tPPI.^7^, ^30^, ^32^, ^38^ However, authors have documented several activities they considered successful, including creative and fun methods (eg visual methods),^30^, ^40^ group work (eg participatory workshops, working groups, group advocacy),^7^, ^35^, ^37^, ^40^ regular meetings,^26^, ^35^ designing products (eg videos, books, tool prototypes),^26^, ^34^ a mail panel,^26^ one‐to‐one discussions,^7^, ^37^ face‐to‐face presentations^34^ and shadowing and mentoring.^34^ It is recommended to formulate goals that can be accomplished in the short term^37^ and to conduct group work separately from adult participants.^26^

#### 3.5.5 Support

Studies have reported the importance of supporting the YPCC involved. YPCC have to learn about, for example, the topic addressed by the project,^9^, ^35^ research methods,^28^, ^34^, ^38^ structures of policy and decision making,^30^, ^35^ and advocacy and communication.^9^, ^35^ Also, they need to be informed about what PPI entails^7^, ^30^ and develop the confidence to share their views.^30^

As such, authors have recommended several ways to support PPI they considered rewarding, including training YPCC,^38^ providing them with feedback^7^, ^30^, ^37^ and appointing experienced facilitators who can inform and support individual YPCC’s.^7^, ^34^, ^37^, ^40^ Creating a comfortable atmosphere in which YPCC feel free to share their experiences^7^, ^37^ and (financially) rewarding them for their involvement^7^, ^29^, ^34^ are also important. Some studies suggested to train professionals in supporting PPI.^7^, ^32^

#### 3.5.6 Power dynamics

In participatory projects, power dynamics are different compared to non‐participatory projects.^28^, ^29^, ^32^, ^38^ Professionals are no longer seen as care provider and project facilitator only, but also as peer.^28^, ^37^ They need to work as a team, with a shared understanding of aims and team members who learn with and from each other.^9^, ^30^

It is, therefore, important that YPCC are respected and taken seriously.^7^, ^35^, ^37^, ^42^ Studies stressed the importance of reassuring YPCC their ideas are valid and important,^7^, ^35^, ^37^ and listening to them and acting on their input.^29^, ^30^ ‘Involvement is more than just listening; it requires follow‐up action’.^30^ This can be challenging, especially when preferences of YPCC, professionals or other stakeholders differ.^35^, ^42^, ^43^ Clear feedback is vital for YPCC to accept that not all their ideas can be taken forward.^7^

#### 3.5.7 Flexibility

PPI requires flexibility and an open mind from professionals.^5^, ^43^ ‘Letting go of controlling the process’, can contribute to the meaningfulness of PPI.^28^ Professionals need to be open to iterative revisions of projects and project materials,^5^, ^40^, ^43^ and, in some cases, to be prepared to go back to square one.^5^ Also, participatory methods need to be flexible, so they can be adapted to the YPCC involved.^38^ This ‘loss of control’ can cause discomfort, when names and reputations are associated with the project.^29^

## 4 DISCUSSION

The aim of this scoping review was to gain insight into the developments in the existing literature on PPI with YPCC in research and implementation projects in health and social care, from the ratification of the UNCRC in 1989. We have mapped reported definitions and goals, activities, experiences and impact.

**Table 3:** Definitions of involvement in the included studies

References	Definition
Brown et al^25^	The term *participative design processes* is used, but no definition is provided.
Bruce and Parker ^9^	*Participatory action research* involves participants for the purpose of addressing a problem that is of sincere concern to them. Integral are self‐determination and advocacy. Four elements are essential: (a) participants identify issues to be studied, (b) participants are directly involved in research, (c) involvement supports individuals in identifying strengths and resources and (d) goal of the research is to improve quality of life
Castensoe‐Seidenfaden et al^26^	*Participatory design* promotes user participation in technology design
Chappell et al^28^	*Participatory research* marks a shift from viewing young people just as ‘objects’. Instead of being bystanders, young people are, to varying levels, engaged in research design, data collection and analysis. Empowerment should be understood as producing ‘alternative power saturated knowledge’ rather than being seen as a commodity to be seized by those perceived as powerles
Chappell^24^	The term *participatory research design* is used, but no definition is provided
Coyne et al^43^	In a *participatory approach,* young people's input is viewed as a central element in design and development. It involves co‐learning and reciprocal transfer of expertise, shared decision making and mutual ownership of processes and products. Four key principles: (a) consultation and cooperation with relevant stakeholders, (b) experimentation with alternative designs, (c) contextualization and (d) iterative development
Flicker^29^	*Community‐based participatory research* is a collaborative approach that equitably involves all partners in research and recognizes everyone's unique strengths. It begins with a research topic of importance to the community with the aim of combining knowledge and action for social change. It is rooted in communities, builds on local knowledge and strengths, directly serves community interests and encourages participation at all levels. It challenges notions of objectivity and the idea that science is apolitical by adopting a set of underlying beliefs and principles that embrace subjectivity
Franklin and Sloper^30^	*Participation* is a continuum along which the type of participation activity should be determined according to the circumstances and the participating children. Levels are being informed, expressing a view, influencing the decision‐making process and being the main decider
Graham et al^31^	The term *advocate* is defined as 'a person who puts a case forward on someone else's behalf'. In this research, it is used to refer to those *involved* in contributing to research projects.
Kramer et al^44^	The project was informed by Lundy's framework that outlines four elements for *participation* in research: (a) ensuring a safe and inclusive space to express views, (b) providing accessible methods to express views, (c) provide an audience and (d) allowing youth to influence decisions
Lightfoot and Sloper^7^	The term *involvement* is used, but no definition is provided
Marshall et al^32^	*Participatory research* seeks to share power between researchers and community participants
McAnuff et al ^33^	The terms *service user involvement* project and of *co‐design* are used, but no definition is provided
Moreau and Eady^34^	The term *community‐based participatory research* is used, but no definition is provided
Murray^35^	The term *participation* is used, but no definition is provided
Powers et al^41^	*Participatory action research* is aimed at involving constituents of research at all levels. It is an approach for bolstering the quality and relevance of research
Rahi et al^36^	The term *child‐centred methods* is used, but no definition is provide
Rich et al^37^	No terms and definitions provided
Rosen‐Reynoso et al^5^	*Youth‐based participatory research* is defined as a collaborative partnership between researchers and youth, to help achieve the project's goals. Similar to community‐based participatory research, it focuses on engaging the youth in all phases of the research, including design, implementation, analysis and dissemination of results
Sloper and Lightfoot^42^	The term *involvement* can encompass a number of different levels of participation from tokenism to children holding increasing power over the content and process of the consultation initiative
Stevenson^38^	*Emancipatory Disability Research* principles indicate that co‐researchers should be involved in data analysis in a way which is meaningful to them; that the data analysis is conducted in a transparent, logical and rigorous manner and aims to produce findings which can be used for the tangible benefit of disabled people. *Participatory Action Research* provided an authentic way in which data can be generated from cycles of planning, acting, observing and reflecting
Van Staa et al^39^	*Patient or user involvement* entails consultation and involvement of patients in all health‐care decisions on the individual and collective level; in the development and evaluation of services; and also in health research. *Participatory research* is a collaborative undertaking aimed at more involvement of the community being studied in all aspects of the research process. It is carried out with and by the research subjects rather than on them. Core elements are co‐learning and reciprocal transfer of expertise, shared decision‐making power and mutual ownership of process and products of the research enterprise
Vindrola‐Padros^40^	The *participatory approach* was guided by four key principles: consultation and cooperation with relevant stakeholders, experimentation with alternative designs, contextualization and iterative development. Inclusion of visual methods in research design does not automatically make it ‘participatory’ as young people's voices might be relegated to the voice of the researcher or other adults or they might not have avenues or the ‘tools’ for shaping the research process

Studies included in the review were published between 2002 and 2017. Most studies were from the last decade, suggesting an increased interest in PPI with YPCC. However, we also found limited progress of the evidence base: definitions continue to be broad and diverse, studies providing high‐quality evidence of the impact are still scarce, and topics addressed remain largely the same. As such, Mayo's^45^ statement that PPI entered the mainstream vocabulary but the practice lagged behind the rhetoric, may—after two decades—still be applicable to the involvement of YPCC.

The challenges of PPI with YPCC are probably an important reason for the limited progress we found in this review. Involvement comes with issues of obtaining additional project resources, representativeness, finding ways to involve YPCC and to support them, dealing with (changing) power dynamics and letting go of controlling the process. Over the years, these challenges continued to exist, with studies reporting similar experiences.

Overcoming challenges related to, for example, changing power dynamics and letting go of the process is fundamental to improving PPI; when professionals resist to share some of their authority and power with YPCC, their involvement will be mostly tokenistic.^18^ This is not easy, as PPI processes are complex and highly dependent on the context they take place in. However, reporting on the same challenges time and again will not help overcome them, but turn them into some impenetrable wall we keep hitting. Therefore, to break down this wall and address the identified challenges, future research should use existing knowledge as a starting point rather than reinvent the wheel.^46^

In line with Bailey et al,^1^ our synthesis has revealed variation in definitions of PPI with YPCC. Studies provide varying definitions—addressing different aspects—for the same terms. Half of the included studies did not provide a definition. Consequently, it is difficult to determine to what extent studies are addressing the same concept.^46^ We have experienced this ourselves during the screening phase of the review, in which we observed that purely qualitative research was sometimes termed ‘participatory’.

Despite the variation in definitions, we also discovered some overlap. We identified five recurring elements, which correspond to elements addressed by Hart's^15^ description of young people's involvement. That is to say, PPI is collaborative (a) and it addresses topics that matter to YPCC (b). It is a continuum of activities providing YPCC with different levels of influence (c) in various stages of a project (d). Finally, PPI is meaningful to both the YPCC involved and YPCC in general (e).

In defining PPI, a trade‐off needs to be made between finding a common definition and allowing for flexibility. When studies use the same words meaning different things—and the other way around—their results become hard to compare. Studies involving YPCC as advisors probably describe different experiences and impact compared with studies involving them as decision‐making partners. At the same time, there may be similarities in experiences and impact, as both studies have involved YPCC in a certain way. Neglecting and poorly defining these differences and similarities causes difficulties in comparing studies and experiences, and in learning about what works for whom and under what circumstances.

However, as Tritter and McCallum have argued,^47^ breadth in defining and operationalizing YPCC’s involvement is also desirable, to allow for flexibility in determining PPI processes. The variety in projects and the way YPCC are involved make formulating one clear and concise definition of PPI difficult. Therefore, we do not promote the use of a strict definition for PPI, but we do urge researchers to include thorough descriptions of what is understood by PPI and how this is translated into activities in future studies, for example, by following the GRIPP‐checklist for reporting PPI.^46^

As in previous reviews,^1^, ^2^ we found few studies providing high‐quality evidence on the impact of PPI. Information on methods and measurements are commonly lacking, and in many cases it is unclear how findings were derived. Also, findings are often based on experiences from those involved in the PPI process themselves, which may have contributed to the predominantly positive experiences in the studies. Also, striking is the shortage of perspectives of YPCC in the reflections on PPI processes. Most findings are based on observations and reflections of professionals. This is remarkable, as PPI by its very nature is about including young people in matters that affect them.^15^

More robust evaluation of PPI with YPCC is needed to convince a broader public of its validity and relevance.^46^ Future research using objective measurements could therefore benefit the evidence base of the impact of PPI and contribute to a more critical discussion. Conducting these measurements is not easy, as PPI is a complex process.^48^, ^49^ Consequently, there is no single method for measuring impact. Studies in which ad hoc consultations were conducted with YPCC should probably adopt different impact assessment criteria and evaluation methods than studies that have extensively collaborated with YPCC as decision‐making partners. One PPI approach may be best evaluated conducting interviews focusing on the benefits for the people involved, while another PPI approach requires observations or questionnaires focusing on the benefits for the project quality. Therefore, we recommend the use of a range of flexible evaluation methods that can be adapted to the project and young people involved.^48^ The authors of a recent literature review have created a useful overview of frameworks for evaluating and reporting PPI that can be applied in different contexts, such as the PiiAF^49^ and the Quality Involvement Framework.^50^

### 4.1 Strengths and limitations

This is the first review to gain insight into the developments in the existing literature on PPI with YPCC by extensively mapping definitions and goals, activities, experiences and impact. We assessed articles published over a broad time period; from the ratification of the UNCRC in 1989 to 2018.

One limitation of this review may be the broad search string on chronic conditions. We may have missed studies focusing on one specific condition. In addition, due to inconsistencies in how involvement is defined and reported, some studies may have been overlooked here as well. To reduce this to a minimum, reference lists of included articles were screened for relevant studies. Another limitation is that we did not include grey literature in the review. These sources may also include relevant information, as it is likely that not all involvement efforts are described in scientific articles.

## 5 CONCLUSION

Based on this review, we recommend conducting more well‐reported research that uses systematic and objective evaluation methods and builds on previous studies to improve the evidence base on PPI with YPCC. There already appears to be a general consensus that YPCC’s involvement should be an integral and standard elements of projects. An improved evidence base can contribute to the validity and reliability of PPI by teaching us about what works for whom and under what circumstances. We also urge YPCC and professionals to just do PPI, despite its challenges. The challenges and complexity of PPI can cause insecurity and a fear of doing it wrong. However, this review has shown that there is not one right method to do it. Moreover, as Lundy^51^ has argued, PPI is seldom perfect; ‘there could always be more time, more resources and more children involved’. When the principles reported in this review are applied (learn from previous work, define and thoroughly report what is meant by PPI, and systematically evaluate its progress and outcomes) valuable lessons can be learned from doing PPI to improve its practice and impact.

## CONFLICT OF INTEREST

No potential conflict of interest was reported by the authors.

## Data Availability

Data sharing is not applicable to this study as no new data were created or analysed.
